# Supplementary figures and images for: A novel genetic circuitry governing hypoxic metabolic flexibility, commensalism and virulence in the fungal pathogen Candida albicans
Source: PLoS Pathog. 2019 Dec 6;15(12):e1007823. doi: 10.1371/journal.ppat.1007823 (PMC6919631; doi:10.1371/journal.ppat.1007823)

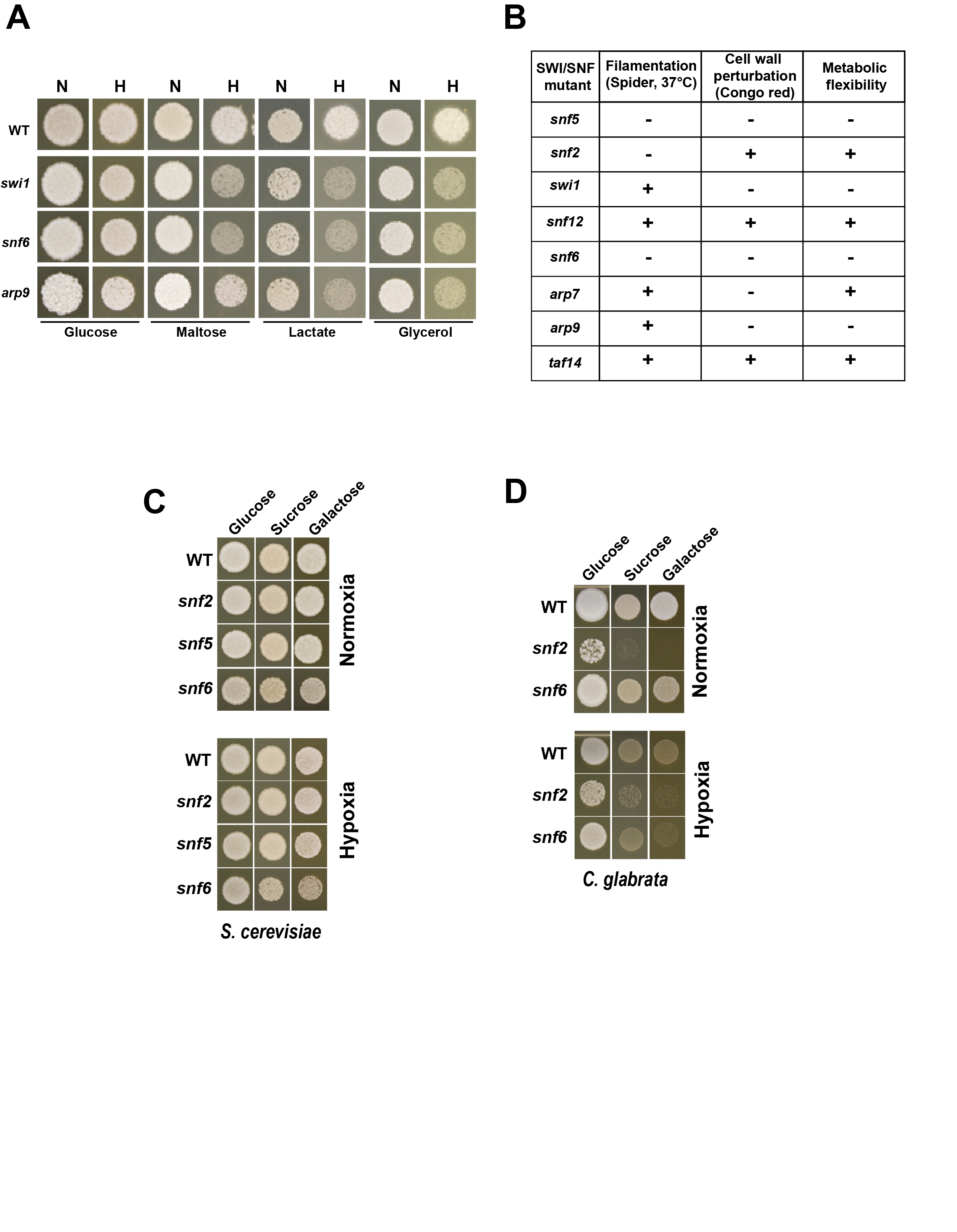

Supplement: S1 Fig — (A) Growth defect of the SWI/SNF subunit mutants swi1, snf6 and arp9 in alternative carbon sources under hypoxia. Mutants were from the GRACE collection and were grown under repressing conditions (100 μg/ml tetracycline) for 4 days at 30°C under both normoxic (N) and hypoxic (H) conditions. (B) Differential requirement of SWI/SNF subunits under conditions that promote hyphal growth or when exposed to the cell wall perturbator, Congo red. The sign + means that a mutation of this gene does not alter sensitivity to Congo red or, have normal morphology and metabolic flexibility. (–) means that a mutation of this SWI/SNF subunit causes hypersensitivity to cell wall stress or, defect in either morphogenesis or metabolic flexibility. (C-D) Metabolic flexibility phenotype of different SWI/SNF subunit mutants of S. cerevisiae (C) and the opportunistic yeast C. glabrata (D). Growth of the WT strain of S. cerevisiae (BY4741) and C. glabrata (HTL) and the SWI/SNF mutants in media with the indicated carbon sources under both normoxic (21% O2) and hypoxic (5% O2) are shown. (TIF) [file ppat.1007823.s001.tif]

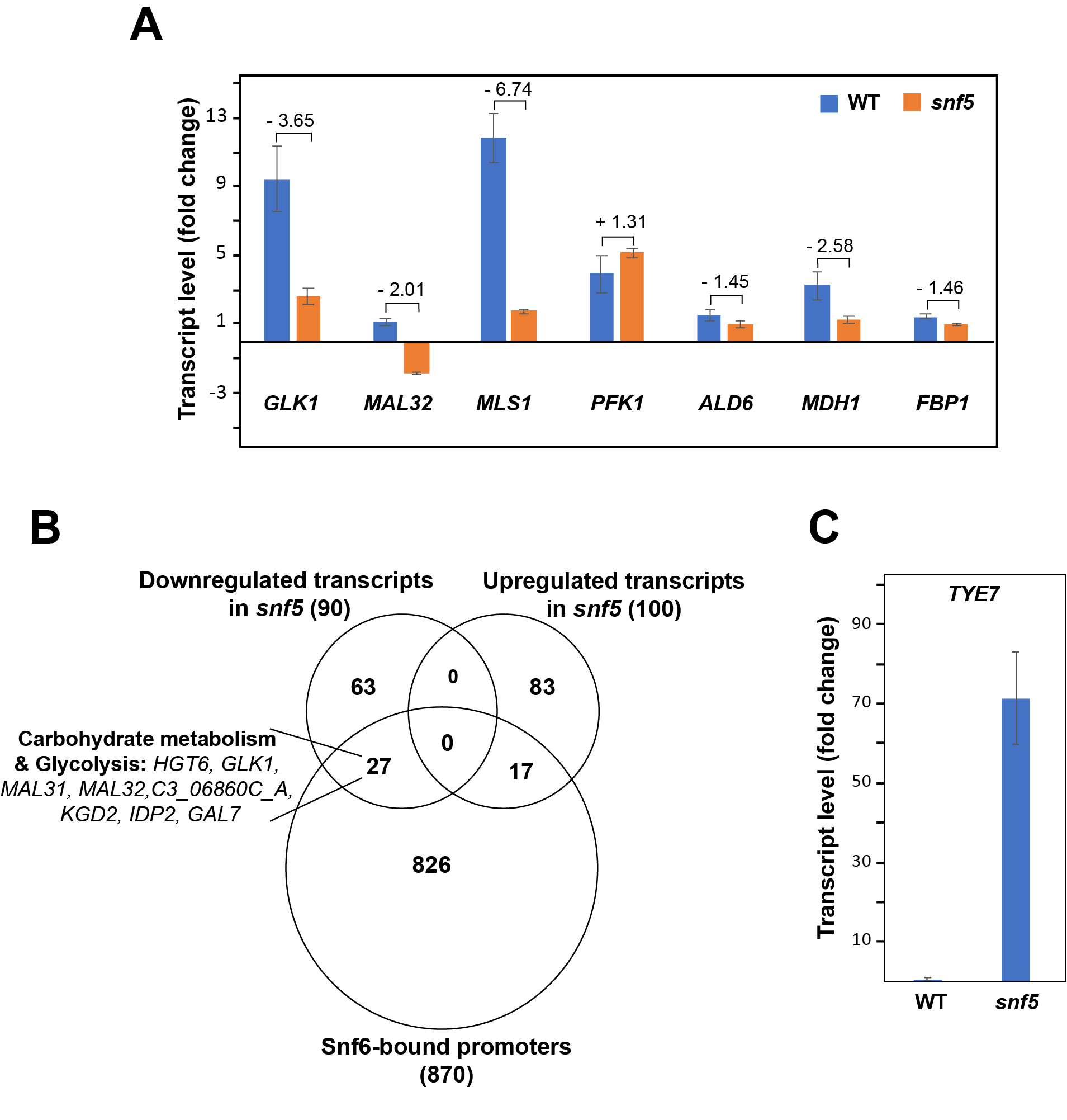

Supplement: S2 Fig — (A) qPCR validation of altered expression levels of GLK1, MAL32, MLS1, PFK1, ALD6, MDH1 and FBP1 in both WT and snf5 mutant strains under hypoxia. Relative expression levels of the seven transcripts were assessed by real-time qPCR and normalized to ACT1 relative to normoxic conditions. Values are the mean from at least two independent experiments. (B) Venn diagram showing overlaps between genes differentially regulated in snf5 mutant and promoters bound by Snf6 as shown by Tebbji et al. [41]. Relevant functional categories are shown. (C) Transcript level of the transcription factor TYE7 in both WT and snf5 mutant strains under hypoxia relative to normoxic conditions. (TIF) [file ppat.1007823.s002.tif]

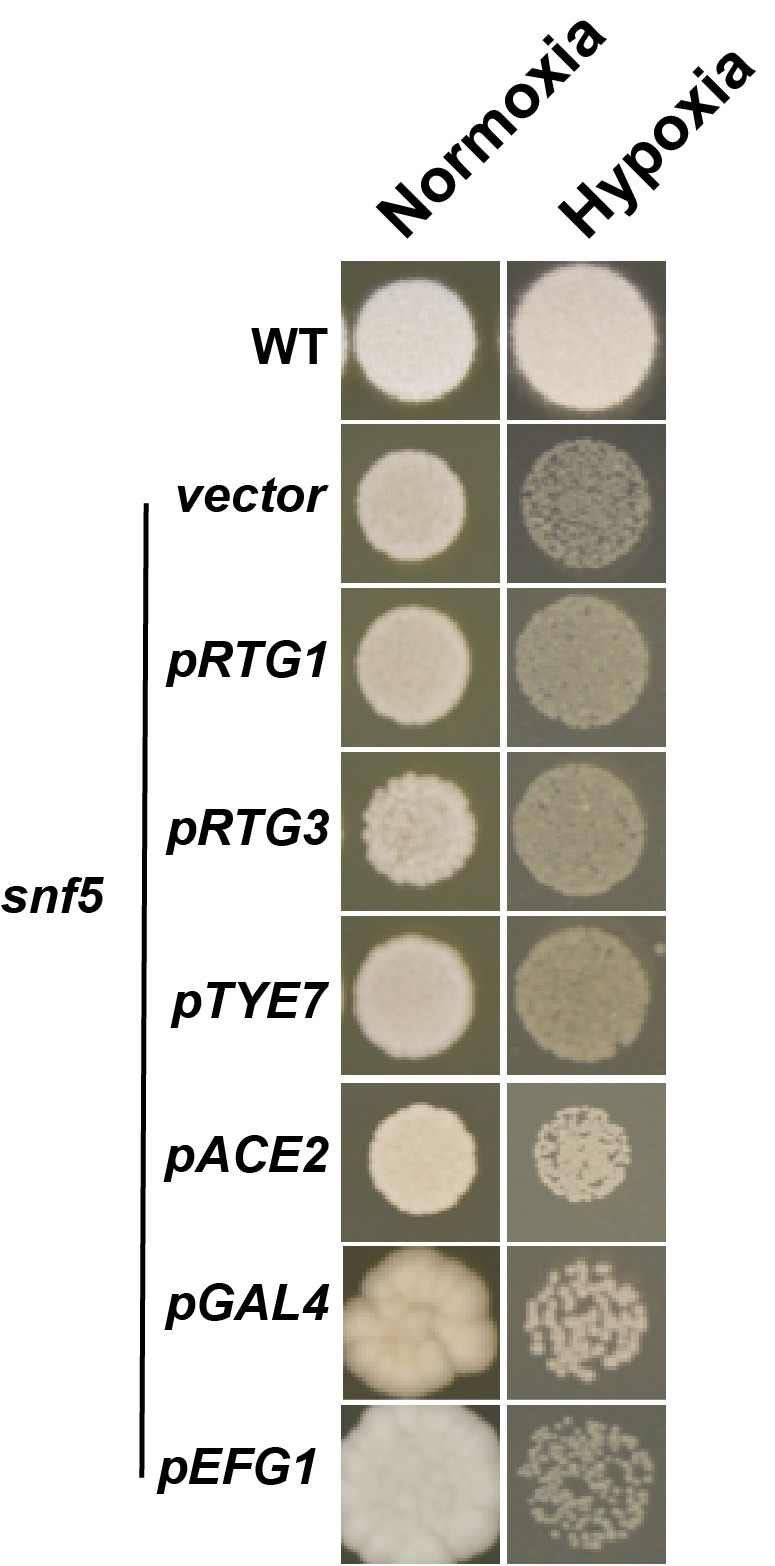

Supplement: S3 Fig — The candidate genes were overexpressed in snf5 mutant and their metabolic flexibility was assessed under both normoxia and hypoxia in YPS medium. (TIF) [file ppat.1007823.s003.tif]
